# Chilaiditi Sign in a Patient With Acute Coronary Syndrome: A Case Report

**DOI:** 10.7759/cureus.36237

**Published:** 2023-03-16

**Authors:** Mina M Guirguis, Jennifer Horawski, Sean L Gibbs

**Affiliations:** 1 Family Medicine, Grand Strand Medical Center, Myrtle Beach, USA

**Keywords:** decreased hepatic size, colonic interposition, diaphragmatic elevation, chilaiditi sign, chilaiditi syndrome

## Abstract

Chilaiditi sign is a radiographic finding where part of the colon is found to be between the diaphragm and liver. Chilaiditi syndrome is characterized by symptoms such as chest or abdominal pain and shortness of breath once Chilaiditi sign is found on imaging. Chilaiditi sign is typically diagnosed by CT angiography (CTA) scan although it can also be seen on X-ray imaging at times. In most cases, Chilaiditi sign does not require acute intervention, as will be seen in our patient; however, it is important to include it in the differential diagnosis when a patient presents with characteristic symptoms. We present a case of a 71-year-old female who presented with chest pressure and shortness of breath due to acute coronary syndrome; however, she was found to have Chilaiditi sign, which was diagnosed by CTA chest.

## Introduction

Chilaiditi sign is defined as the interposition of bowels between the liver and right diaphragm [1]. This finding is seen in 0.025-0.28% of the population and typically involves the hepatic flexure or transverse colon [1]. The term “Chilaiditi sign” was coined by the Greek radiologist Demetrius Chilaiditi in 1910; however, when this sign is found in symptomatic patients, it is called Chilaiditi syndrome [1]. Symptoms of this syndrome include abdominal pain, nausea, vomiting, respiratory distress, constipation, and obstruction. Often, this disease is found incidentally. The etiology of Chilaiditi syndrome can be congenital or acquired [2]. This can include the absence of dispensary or falciform ligaments, malrotation, and paralysis of the right diaphragm [1]. While the cause is often unknown, the treatment of Chilaiditi sign varies according to the clinical picture of the patients and whether they are symptomatic, which is quite unusual.

## Case presentation

A 71-year-old female presented to the emergency department with a two-day history of progressive shortness of breath after a coughing spell. She reported occasionally having these coughing spells, which would always self-resolve; however, this time it progressed to dyspnea on exertion with an associated substernal chest pressure without any radiation. She denied any fevers, chills, syncopal episodes, or previous history of myocardial infarction.

On admission, she appeared uncomfortable but was inquiring whether she would need to stay the night. Her pulse was 114 beats per minute, and her BP was 148/71 mmHg. Her breathing requirements also worsened. Her respiratory rate was 21 breaths per minute and she had an oxygen saturation of 94% on 4 liters of nasal cannula. No jugular venous pressure or cardiac murmurs were appreciated on the exam. She did not appear to have decreased breath sounds but admitted to having minor difficulty with breathing. Her abdomen was soft and non-tender and had normal bowel sounds.

**Figure 1:**
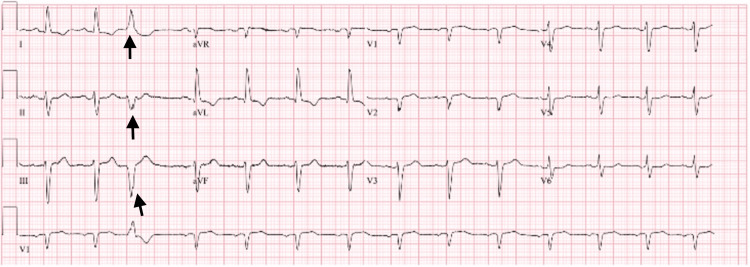
Initial EKG revealing nonspecific premature ventricular complexes (PVCs) EKG: electrocardiogram

**Figure 2:**
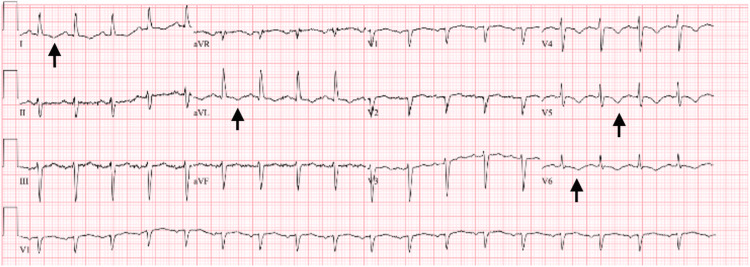
Notable T-wave changes in the lateral leads

A series of electrocardiograms were performed. The first one showed a sinus rhythm with occasional premature ventricular complexes (Figure 1). Later that evening, she was found to have a left anterior fascicular block with possible septal infarcts and T-wave changes indicating possible lateral ischemia (Figure 2).

**Figure 3:**
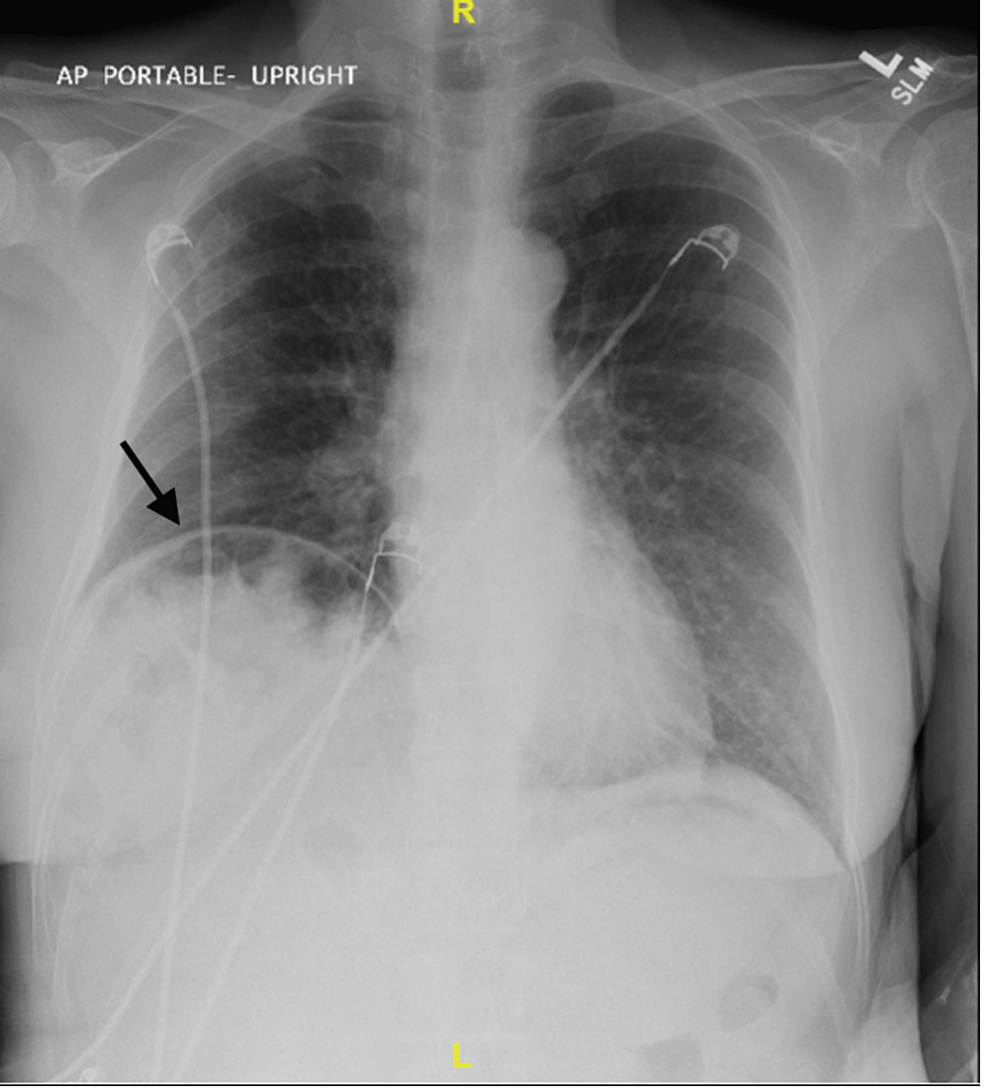
Marked elevation of right hemidiaphragm

**Figure 4:**
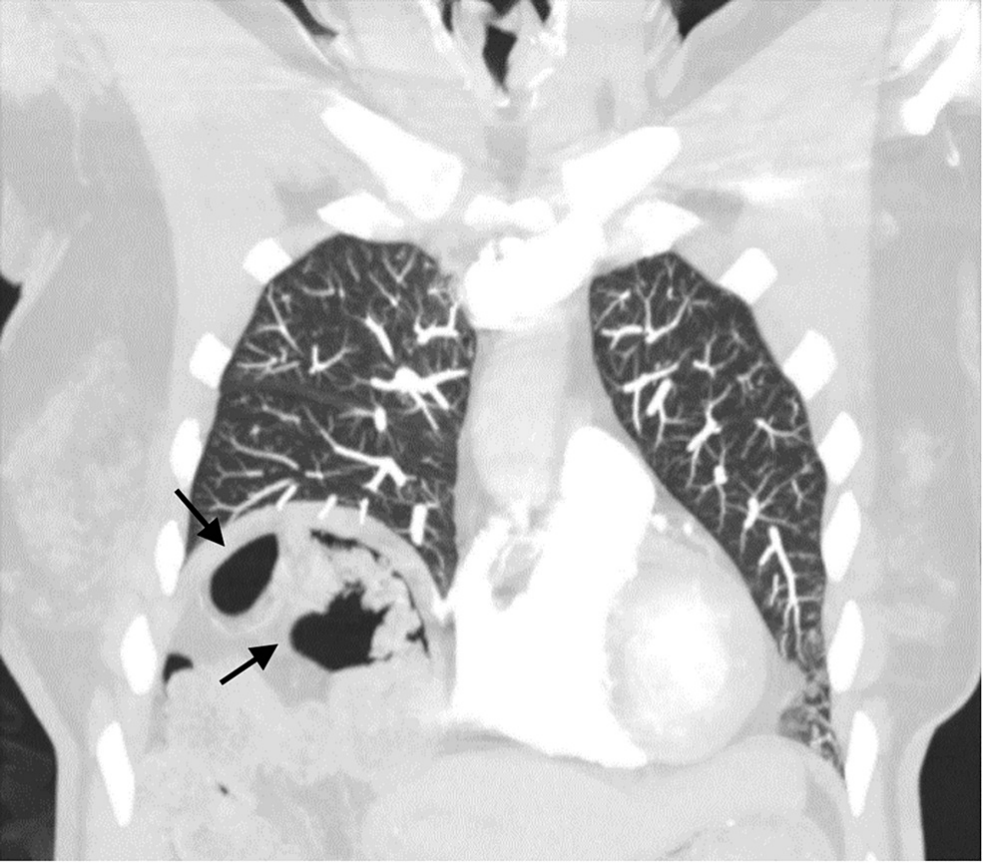
Consistent elevation of right hemidiaphragm with colonic interposition and mild stool burden

The chest radiograph showed marked elevation of the right diaphragm with colonic interposition, as seen in Figure 3. CT angiography (CTA) of the chest and thorax revealed bibasilar atelectasis on the right as well as colonic hepatic interposition and right hemidiaphragmatic elevation with mild colonic stool burden (Figure 4).

The patient’s workup began with basic laboratory investigations. Notably, her cardiac enzymes were elevated. The initial troponin-I level was 1.16 ng/ml (normal range: 0.0-0.04 ng/ml) with the repeat test showing a level of 0.875 ng/ml. The basic metabolic panel was notable for a potassium of 3.3 mEq/L (normal range: 3.50-5.20 mEq/L) but was otherwise within normal limits. Complete blood count with differential was remarkable for a white blood cell count of 16.500 mm^3^ (normal range: 4.500-11.000 mm^3^). Notably, the patient’s hemoglobin A1c was 5.5% (normal range: less than 6.5%), but her LDL was 136 mg/dl (normal range: less than 100.00 mg/dl). Thyroid and kidney function tests were within normal limits.

She was managed with intravenously administered fluids, and was loaded on aspirin 324 mg, started on atorvastatin 40 mg, a heparin drip, sublingual nitroglycerin, oxygen, and further pain control medications as needed. The patient was taken for left heart catheterization later that evening, which revealed severe left main and severe three-vessel coronary artery disease in the setting of normal left ventricular function and accelerating anginal symptoms.

The next day, the patient underwent coronary artery bypass grafting with cardiothoracic surgery, and her chest pain and shortness of breath subsequently resolved. One day later, she was deemed medically stable after further observation for discharge home. She was informed about the results of the imaging studies, and she chose to be discharged home once the pain subsided. She was encouraged to follow up if her shortness of breath worsened, or if she started to experience new-onset symptoms including abdominal pain, nausea, vomiting, and decreased stool output.

## Discussion

We discussed the case of a patient who presented with chest pressure and dyspnea on exertion and was found to have bowel in her chest cavity on imaging. Diaphragmatic injury is uncommon, representing only about 1% of all traumatic injuries [3]. A high index of suspicion must be maintained because a delayed diagnosis can lead to poor outcomes, including herniation and strangulation of abdominal organs. However, it must be highlighted that Chilaiditi sign can also lead to a false-positive diagnosis of diaphragmatic injury.

As described, Chilaiditi sign is the radiologic finding whereas Chilaiditi syndrome is the clinical manifestation. The etiology of Chilaiditi syndrome, often unknown, can be congenital or acquired [2]. This includes the absence of dispensary or falciform ligaments, malrotation, and paralysis of the right diaphragm [1]. Although an extremely rare disease, a common cause is thought to be chronic obstructive pulmonary disease (COPD). With the elevation of the lower thoracic cage, there is more space available for colonic interposition to occur. Furthermore, diseases that tend to increase the space between the liver and diaphragm, such as cirrhosis and overall decreased hepatic size, may further trigger Chilaiditi sign/syndrome.

Patients with Chilaiditi syndrome may experience symptoms of anorexia, nausea, vomiting, abdominal pain, and distention. Similarly, these symptoms may be seen in many differential diagnoses, notably an acute abdomen or a bowel obstruction. Therefore, it is important that we evaluate the patient as a whole. Given that the signs and symptoms of Chilaiditi syndrome often mirror those of bowel obstruction, the diagnosis is made based on radiographic imaging. Close inspection of an abdominal X-ray film, specifically in the left lateral decubitus position, may reveal colonic haustra under the right diaphragm [4]. However, plain film X-rays are often nonspecific [5]. In most cases that raise suspicion, a CT scan is now the investigation of choice. This is because it not only helps confirm findings of bowel under the diaphragm but also helps differentiate the condition from potential life-threatening diagnoses such as acute abdomen, pneumoperitoneum, and subphrenic abscesses [6].

The treatment of Chilaiditi syndrome often varies based on the patient’s clinical picture. In our case, the patient was diagnosed with acute coronary syndrome while Chilaiditi sign was an incidental finding on the CTA chest. However, in symptomatic cases, the management is often conservative and consists of bowel rest, decompression, and fluid hydration [7,8]. Patients who fail conservative management should undergo exploratory laparotomy to avoid possible complications including both colonic volvulus and obstruction, herniation, and strangulation [9]. Moreover, it is important to note that counseling patients regarding lifestyle modifications including a healthy diet, exercise, and routine bloodwork to monitor their lipids and blood sugar levels is important in the prevention of Chilaiditi syndrome [10].

## Conclusions

Chilaiditi syndrome is characterized by symptoms such as shortness of breath once Chilaiditi sign is found on imaging. Chilaiditi sign, typically diagnosed by a CTA scan, is a finding where a part of the colon is found to be interposed between the diaphragm and liver. Although asymptomatic in most cases, it is important to keep it in mind when patients present with clinical signs and symptoms of pneumoperitoneum. The diagnosis can often be challenging. This case report highlights the importance of evaluating the individual as a whole and not just their imaging or lab findings, thereby preventing unnecessary tests and poor outcomes.
